# 

**Published:** 1874-12

**Authors:** 


### INDEX.

A PAGE

Abortion ... 127
Abortion, Criminal—Dr. J. S. Whitmire, 385
Abortion not a Crime ... 569
Adolphus, Dr. Philip, Gynæcology and Obstetrics ... 625
A Grateful Tribute ... 57
Ague Plant—Dr. Jno. Bartlett ... 1
Agnew, D. Hayes, M.D., Lacerations of the Female Perineum ... 54
Alcohol, Physiological Influence of ... 113
American Medical Association ... 317
Ascites, Case of—J. M. West, M.D ... 451
Auscultation, Fœtal, Fifty cases reported —Dr. A. B. Strong ... 524

B

Baldwin, M. O., M.D., New Treatment for Burns ... 469
Bartlett (Dr. Jno.) on a Marsh Plant from the Mississippi River Ague Bottoms, 1
Bladder, Perforation of—Dr. L. J. Willien ... 460
Bladder and Rectum, Use of Ice in—Dr. H. M. Lyman ... 516
Barker, Puerperal Diseases ... 372
Bloodless Surgery—J. E. Kelly, M.D ... 105
Board of Health ... 126
Bogus Diploma Traffic ... 61
Book-Keeping, Treatise on — Dr. J. Adams Allen ... 434
Book Notices—
Allport, Address delivered before the American Academy of Dental Science ... 754
American Journal of Obstetrics, etc ... 437
Annual Report of Surgeon of U. S. Marine Hospital ... 371
Barnes, Surgical Diseases of Women ... 374
Beattie, Dr. W. F. C., Recovery from Bite of a Rattlesnake ... 51
Bellamy, Students' Guide in Surgical Anatomy ... 312
Brodie, Mind and Matter ... 120
Bull and Hansen, Leprous Diseases of the Eye ... 122
Burns, New Treatment for—M. Olmsted Baldwin, M.D ... 469
Chicago Medical Register and Directory ... 746
Davis, Clinical Lectures on Various Subjects ... 748
Elmer, Physicians' Hand Book ... 52
Farabœuf, Ligation of Arteries ... 748
Fenwick, Students' Guide to Medical Diagnosis ... 53
Flint, Physiology of Man ... 627
Griffith, Formulary ... 565
Hammond, Nervous System ... 697
Hartshorne's Conspectus of Medical Sciences ... 501
Howe, Manual of Eye Surgery ... 753
Jacobi, Infant Diet ... 746
Lincoln, Electro-Therapeutics ... 502, 747

Book Notices— PAGE
Marvin, Philosophy of Spiritualism—Butts ... 751
Minor, Erysipelas and Child-bed Fever 754
Montgomery, W. T. T., M.D., Typhoid Fever—Collation of 112 Cases. 577
Murray, Pathology and Treatment of Children ... 564
Parrish, Treatise on Pharmacy ... 438
Peugnet, The Nature of Gunshot Wounds of the Abdomen ... 750
Physicians' Pocket Case Record ... 52
Physicians' Visiting Lists, 1874 ... 52
" " " for 1875 ... 750
Reese, Manual of Toxicology ... 696
Reynolds' Lectures on the Clinical Uses of Electricity ... 312
Ringer, Dr. Sydney, On Rheumatic Fever ... 49
Roberts, Theory and Practice of Medicine ... 120
Rockwell, A. D., A.M., and Geo. M. Beard, M. D., Clinical Researches in Electro-Surgery ... 311
Schrœder, Manual of Midwifery ... 52
Swain, Surgical Emergencies ... 749
Thomas, Diseases of Women ... 697
Thompson, Sir Henry, On Calculous Disease ... 54
Van Buren, Surgical Diseases of Genito-Urinary Organs ... 563
West, On Diseases of Infancy and Childhood ... 440
Wood, Treatise on Therapeutics ... 628
Wyeth, A Hand-Book of Medical and and Surgical Reference ... 179
Wythe's Physicians' Dose and Symptom Book ... 313
Bowman, Practical Chemistry ... 121
Brain and Nervous System—Dr. W. Hay, 723
Bridge, Norman, M.D., Dislocation of the Ulna ... 147
Trichinosis ... 233
Progress in Practice of Medicine ... 4c8
Stammering, Loss of Voice, and Neuralgia from Constipation ... 431
Progress in Practice of Medicine ... 611

C

Carcinoma Uteri ... 127
Central Illinois Medical Society, Transactions of ... 243
Cerebro-Spinal Meningitis — James S. Whitmire, M.D ... 257
Chicago Soc. Physicians and Surgeons ... 173
Chicago Mortality Reports—
By Dr. Ben. C. Miller.
For January, 1874 ... 189
For February, 1874 ... 254
For April, 1874 ... 381
For May, 1874 ... 445
For June, 1874 ... 510
For July, 1874 ... 574
For August, 1874 ... 638

PAGE
Chicago Mortality Reports—
For September, 1874 .......... 702
For October, 1874 .......... 760
Cholera, Asiatic, Facts and Theories About—By Dr. J. C. Peters .......... 44
Cholera .......... 572
Chorea .......... 128
Clinics—
Hay, Dr. Walter .......... 723
Powell, Dr. E .......... 717
Dr. Gunn, September, 1874 .......... 725
Consistency .......... 572
County Hospital .......... 435
Correspondence .......... 701
Criminal Abortion—J. S. Whitmire, M.D. 385
Crowner's 'Quest .......... 62

D

Dalby, Diseases and Injuries of the Ear, 121
Danforth, I. N., M.D., Pathology and Microscopy .......... 328
Dawson, B. F., Vascular Nævi treated with the Galvanic Cautery .......... 470
Declined .......... 434
DeWolf, O. C., M.D., Impermeable Urethral Stricture .......... 393
Koumiss, and its Use in Medicine .......... 658
Digitalis, Is it a Cardiac Sedative, or Cardiac Stimulant?—T. Curtis Smith M.D .......... 734
Dignity of the Profession .......... 378
Dispensary, Central—Dr. N. Bridge .......... 431
Disinfectants .......... 627
Directory for May, 1874 .......... 320
For June, 1874 .......... 377
For July, 1874 .......... 448
Dunglison's Medical Dictionary .......... 176

E

Electricity, Physiological Action of Induced Currents .......... 344
Elixirs .......... 184
Emery, G. W., M.D., Habitual Regurgitation .......... 450
Thoughts on Dr. Whitmire's Paper on Criminal Abortion .......... 598
Ergotine, Hypodermic Use of, in Uterine Tumors—Dr. J. H. Etheridge... 360
Epilepsy, Case of—Walter Hay, M.D... 369
Expert Testimony—Thad. M. Stevens, M.D .......... 117
Etheridge, J. H., M.D., Progress in Therapeutics .......... 337
Hypodermic Use of Ergotine in Uterine Tumors .......... 360
Evans' History of the American Ambulance .......... 439

F

Femur, Fracture of—J. E. O'Brien, M.D. 450
Fever, Rheumatic, treated by a cold water bath .......... 49
Fever, Typhoid, Collation of 112 Cases —W. T. T. Montgomery, M.D .......... 577
Fiat Justitia Ruat Cœlum .......... 437
Fibrous Tumors of the Uterus — A. Reeves Jackson, M.D .......... 350
Fisher, Dr. S., Retroversion of the Uterus .......... 362
Fistula, case of Vesico-Vaginal—J. E. O'Brien, M.D .......... 591
Fœtal Auscultation—Dr. A. B. Strong.. 524
Freer, Dr. Jos. W., Historical Sketch of Rush Medical College .......... 187

PAGE
Freer, Dr. Jos. W., Transfusion of Blood, 427
Surgical Clinic .......... 692

G

Gastralgia .......... 127
Gorton's Mental Hygiene .......... 118
Graham, D. W., M.D., Case of Habitual Regurgitation .......... 326
Gynæcology, Report on — A. Reeves Jackson, M.D .......... 149
Gynæcology and Obstetrics—Service of Dr. Philip Adolphus .......... 625
Gunn, Moses, M.D., Surgical Clinic, 560, 725

H

Hemorrhage from Internal Carotid Artery treated by Ligature .......... 108
Hay, Walter, M.D., Myelitis .......... 207
Neuro-Pathology and Pyschological Medicine .......... 215
Case of Epilepsy .......... 369
Three Cases of Progressive Muscular Atrophy .......... 429
Clinic for Neuro-Pathology .......... 623
Clinic for Diseases of the Nervous System .......... 690
Diseases of the Brain and Nervous System .......... 723
Hayes, Justin, Multilocular Sero-cystic Ovarian Tumor treated .......... 517
Hayes, P. S., M.D., Report of Chicago Soc. Physicians and Surgeons .......... 94
Hays, J. L., M.D., Report on Surgery.. 453
Head, Injury of the—Geo. J. Monroe, M.D .......... 665
Hernia, Case of Strangulated—J. W. Robbins, M.D .......... 524
Holden, Sphygmograph .......... 176
Homicide, Suspected Simulation of Insanity—I. Ray, M.D .......... 726
Homocultology .......... 116
Hospital Reports—
Alexian .......... 477
Central Free Dispensary .......... 625
Cook County .......... 427, 473, 557, 692, 717
Mercy .......... 560
St. Joseph's .......... 480
St. Luke's .......... 694
Woman's, of State of Illinois—W. C. Lyman, M.D .......... 228
Hyde, James Nevins, M.D., Progress of Syphilology .......... 303, 533
Progress of Syphilology and Dermatology .......... 398
Sex and Contagion .......... 642
Hymen, Complete Closure of—G. C. Paoli, M.D .......... 328

I

Ice, Use of, in Painful Conditions of the Bladder and Rectum—H. M. Lyman, M.D .......... 516
Illinois State Prison .......... 182
Imbeciles, Asylum for .......... 249
Impermeable Urethral Stricture — O. C. DeWolf, M.D .......... 393
Infants, Their Food and its Digestion—A. S. von Mansfeldt, M.D .......... 129
Infants and Their Food—G. Newkirk, M.D .......... 284
Infants, Food For, A Review of the Review—Dr. A. S. von Mansfeldt .......... 462
Infants and Their Food, Summary .......... 594

### *Index.*

PAGE

Insanity, Suspected Simulation of—Dr. I. Ray ... 726
Intestines, Extensive Distension with Gas—C. T. Parkes, M.D ... 143

**J**

Jackson, A. Reeves, M.D., Obstetrics and Diseases of Women ... 74
Report on Gynæcology ... 149
Obstetrics and Gynæcology, Report on, 220
Obstetrics and Gynæcology ... 333
Fibrous Tumors of the Uterus ... 350
Obstetrics and Gynæcology, Progress in ... 600
Johnson, Lectures on Bright's Disease, 314

**K**

Kohlrausch, Dr. F., Physical Measurements ... 502
Koumiss, and its Use in Medicine—O. C. DeWolfe, M.D ... 658

**L**

Labor, Puerperal Fever and Death—G. J. Monroe, M.D ... 145
Law *vs.* Physic ... 185
Lee, E. W., M.D., Pneumatic Aspirator, 331
Lincoln, D. F., Electro-Therapeutics, 502, 747
Loot ... 127
Lunatics, Are they Criminals? ... 125
Lyman, W. C., M.D., Woman's Hospital of Illinois, Report of ... 228
Lyman, Dr. H. M., on the use of Ice in Painful Conditions of the Bladder and Rectum ... 516

**M**

McElroy, Z. C., Review of The Chicago Medical Journal for April, 1874 ... 290
Malarial Diseases—By Dr. Jno. Bartlett, 1
Mansfeldt, A. S. von, M.D., Infants, Their Food and its Digestion ... 129
Food for Infants ... 462
Marsh Plant, from the Mississippi River Ague Bottoms ... 1
Medico-Historical Society ... 379
Meigs' Diseases of Children ... 371
Miller, DeLaskie, M. D., Valedictory Address ... 194
Answer to certain Criticisms ... 381
Mitchell, A., M.D., Bite of a Rattlesnake ... 42
Monroe, Dr. Geo. J., Labor, Puerperal Fever and Death ... 145
Case of Injury to the Head ... 655
Monstrosity, Case of—F. E. English, M.D ... 213
Mortality Statistics ... 186
Muscular Atrophy, Three Cases of—Walter Hay, M.D ... 429
Myelitis—Walter Hay, M.D ... 207

**N**

Nervous System, Clinic for Diseases of—Walter Hay, M.D ... 690
Neuro-Pathology and Psychological Medicine—Walter Hay, M.D ... 215
Neuro-Pathology, Clinic for—Walter Hay, M.D ... 623
Newkirk, G., M.D., Review of the Article, "Infants and Their Food," ... 284
Not so Black as Painted ... 758

**O** PAGE

Obituary—
Bucher, Dr. S. R ... 702
Holston, Sr., Dr. J. G. F ... 370
Parker, Dr. Milton ... 64
Woodward, Joseph ... 64
O'Brien, J. E., M.D., To Prevent Rotation in Fracture of the Femur ... 450
Case of Vesico-Vaginal Fistula ... 591
Obstetrical Cases, Observations in—Dr. D. A. K. Steele ... 530
Obstetrics and Gynæcology—A. Reeves Jackson, M.D ... 220, 333
Obstetrics and Diseases of Women—Dr. A. Reeves Jackson ... 74
Obstetrics and Gynæcology, Progress in—A. Reeves Jackson, M.D ... 600
One More Unfortunate ... 506
Ovarian Tumor Treated—Justin Hayes, 517
Owens, Dr. Jno. E., Staphyloraphy and Uranoplasty ... 65
Progress of Surgery, 153, 224, 297, 414, 605, 705

**P**

Paoli, G. C., M.D., Complete Closure of Hymen ... 328
Pathology and Microscopy—I. N. Danforth, M.D ... 328
Peripheral Paralysis—Dr. S. G. Webber, 96
Paraplegia, Case of—G. W. Stewart, M.D ... 209
Parasitic Origin of Disease—E. A. Parkes, M.D ... 161
Parkes, Dr. C. T., Intestines, Extensive Distension of ... 143
Pavy, Food and Dietetics ... 694
Periodical International Congress of the Medical Sciences ... 507
Peters, Dr. J. C., on Asiatic Cholera ... 44
Philadelphia University of Medicine ... 248
Placenta, Remarkable Retention of—Dr. C. E. Ristine ... 522
Pneumatic Aspirator employed successfully—E. W. Lee, M.D ... 331
Powell, Prof, E., Surgical Clinic ... 717
Practice of Medicine, Progress in—Norman Bridge, M.D ... 408, 611
"Preparatory Medicines," ... 758
Progress in Medical Sciences, 149, 215, 328, 398, 533, 601
Progress of Surgery—Jno. E. Owens, M.D ... 153, 224, 297, 414, 605, 705
Publishers' Notice ... 572

**Q**

Quarantine and Public Health ... 634

**R**

Rattlesnake, Bite of—Dr. A. Mitchell .. 42
Rattlesnake, Recovery from Bite of a—Dr. W. F. C. Beattie ... 51
Ray, I., M.D., Homicide, Suspected Simulation of Insanity ... 726
Reeder, J. N., M.D., Case of Strangulated Umbilical Hernia ... 323
Regurgitation, Habitual—G. W. Emery, M.D ... 450
Regurgitation, Habitual—D. W. Graham, M.D ... 326
Retroversion of Uterus—Dr. S. Fisher .. 362
Review of The Chicago Medical Journal for April, '74—Z. C. McElroy, M.D., 290

PAGE

Rheumatic Fever, treated with a Cold Bath—Dr. Sidney Ringer........... 49
Ristine, Dr. C. E., Remarkable Case of Retention of Placenta............... 522
Robbins, J. W., M.D., Case of Strangulated Hernia reduced by Gravitation and Traction........................ 524
Rush Medical College............ ..... 61
Historical Sketch, by President Freer, 187
Valedictory Address, by DeLaskie Miller, M.D ........................ 194
Names of Graduates, 1873-4.......... 319
Clinic of Nervous Diseases—Dr. Walter Hay................ ............ 479
Surgical Clinic—By Moses Gunn, M.D. 560
Clinics.................................. 623
Disease of the Brain and Nervous System—Dr. Walter Hay............ .. 723

S

Salivation................................ 127
Sanitary Science......................... 248
Sanitary ................................. 701
Sex and Contagion—Dr. James Nevins Hyde................................. 642
Sex in Utero—Dr. D. A. K. Steele...... 530
Skin Grafting ... ..................... ... 128
Smith, T. Curtis, M.D., Digitalis, Is it a Cardiac Sedative or Cardiac Stimulant? .. ...... ................ 734
Sponges................................ 631
Staphyloraphy and Uranoplasty—Jno. E. Owens, M.D.................... 65
Steele, Dr. D. A. K., Record of Fifty Obstetrical Cases.................... 530
Strangulated Umbilical Hernia—J. N. Reeder, M.D........ ........... 323
Strong, Dr. A. B., Fœtal Auscultation.. 524
Stewart, G. W., M.D., Case of Paraplegia..... ............................. 209
Surgery, Bloodless—J. E. Kelly, M.D.. 105
Surgery, Progress of—Jno. E. Owens, M.D..... .....153, 224, 297, 414, 605, 705
Surgery, Report on—J. L. Hays, M.D... 453
Surgical Clinic—Dr. Freer.............. 692
Syphilology, Progress of—James Nevins Hyde, M.D.....................303, 533
Syphilology and Dermatology, Progress of—James Nevins Hyde, M.D...... 398

T

Therapeutics, Progress in—J. H. Etheridge, M.D......................... 337

PAGE

Transfusion of Blood—Dr. Freer....... 427
Trichinosis—Norman Bridge, M.D...... 233
Typhoid Fever, Collation of 112 Cases —W. T. T. Montgomery, M.D..... 577
TRANSACTIONS—
Æsculapian Society of the Wabash Valley ............................ 496
Central Illinois Medical Society...... 243
Chicago Medico-Historical Society... 571
Constitution and By-Laws of....... 425
Chicago Medical Society, 366, 421, 481, 553, 677
Chicago Society of Physicans and Surgeons, 94, 237, 252, 348, 422, 489, 544, 615, 667, 709
Abstract of Proceedings of, for 1873.. 252

U

Ulna, Dislocation of—Norman Bridge, M.D......... ................ ....... 147
Umbilical Hernia, Case of Strangulated —J. N. Reeder, M.D ........... 323
Uterine Hemorrhage—L. J. Willien, M.D 457
Uterine Tumors, Hypodermic Use of Ergotine in—Dr. J. H. Etheridge... 360
Uterus, Fibrous Tumors of the—A. Reeves Jackson, M.D.... .. ...... 350
Uterus, Retroversion of—Dr. S. Fisher.. 362

V

VanBuren, Practical Treatise on the Surgery of the Genito-Urinary Organs. 442
Vascular Nævi treated with Galvanic Cautery—B. F. Dawson, M.D...... 470
Vesico-Vaginal Fistula — Dr. J. E. O'Brien............................ 591
Vital Statistics, Compilation of....... . 759

W

Waxy Kidney—M. W. Wood, M.D..... 273
Webber, Dr. S. G., Peripheral Paralysis, 96
West, J. M., M.D., Case of Ascites treated ............................. 451
Whitmire, Dr. J. S., Meningitis or Spotted Fever...... . ................. 257
Criminal Abortion.................... 385
Who is a Regular Physician?........... 634
Who is He?....... ............. ....... 377
Willien, L. J., M.D., Uterine Hemorrhage 457
Case of Perforation of the Bladder.... 460
Wilson, The Anatomist's Vade Mecum . 178
Wood, M. W., M.D., Waxy Kidney.... 273

THREE DOLLARS A YEAR. SPECIMEN COPIES, 30 CENTS.

VOL. XXXI. December, 1874. NUMBER 12.

THE

### Chicago Medical Journal.

J. ADAMS ALLEN, M.D., LL.D., WALTER HAY, M.D.,

EDITORS.

TWELVE TIMES A YEAR.

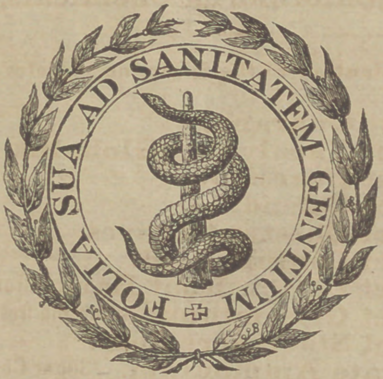

### CONTENTS:

PROGRESS IN MEDICAL SCIENCES:

ART. I. Progress of Surgery. By Jno. E. Owens, M.D., Chicago ..... 705

REPORTS OF SOCIETIES:

Chicago Society of Physicians and Surgeons ..... 709

HOSPITALS:

Cook County Hospital. Dr. Powell ... 717

CLINICS:

Rush Medical College. Drs. Hay and Gunn ..... 723

SELECTIONS:
Homicide—Suspected Simulation of Insanity ..... 726
Digitalis—Is it a Cardiac Sedative, or Cardiac Stimulant? ..... 734

EDITORS' BOOK TABLE ..... 745

EDITORIAL ..... 758

MORTALITY STATISTICS ..... 760

**TERMS, ALWAYS IN ADVANCE:** One copy, per annum.... $3 00

☞ Yearly subscriptions must begin with the January number.

☞ Persons who send subscriptions for the JOURNAL will please consider the first number received after subscription as a sufficient receipt for money sent.

☞ We cannot return rejected manuscripts.

CHICAGO:

W. B. KEEN, COOKE & CO., PUBLISHERS,

NOS. 113 AND 115 STATE STREET.

☞ **Back Nos. for 1872, '73 and '74 can be supplied at 30 cents each.**

